# NEDD4L inhibits cell viability, cell cycle progression, and glutamine metabolism in esophageal squamous cell carcinoma via ubiquitination of c-Myc: NEDD4L and c-Myc coordinate to regulate ESCC

**DOI:** 10.3724/abbs.2022048

**Published:** 2022-05-16

**Authors:** Wei Cheng, Guiyuan Li, Zhou Ye, Jun Hu, Lixia Gao, Xiaoling Jia, Suping Zhao, Yan Wang, Qin Zhou

**Affiliations:** 1 Department of Hematologic and Oncology Xinjiang Clinical Research Center for Precision Medicine of Digestive System Tumor the Center Hospital of Karamay City Karamay 834000 China; 2 Department of Oncology Tongji Hospital School of Medicine Tongji University Shanghai 200065 China; 3 Department of General Surgery Xinjiang Clinical Research Center for Precision Medicine of Digestive System Tumor the Center Hospital of Karamay City Karamay 834000 China; 4 Department of Science and Education the Center Hospital of Karamay City Karamay 834000 China

**Keywords:** esophageal squamous cell carcinoma, glutamine metabolism, NEDD4L, ubiquitination

## Abstract

Esophageal squamous cell carcinoma (ESCC) is a common subtype of esophageal cancer with high incidence. Surgery remains the main strategy for treatment of ESCC at early stage. However, the treatment outcome is unsatisfactory. Therefore, finding new therapeutics is of great importance. In the present study, we measured the level of NEDD4L, an ubiquitin protein ligase, in clinical samples and investigated the effects of NEDD4L on cell viability, cell cycle progression, and glutamine metabolism in TE14 cells determined by CCK-8 assay, flow cytometry and biochemical analysis, respectively. The results show that NEDD4L is significantly decreased in ESCC specimens, and its decreased expression is associated with a poor clinical outcome. Overexpression of NEDD4L significantly inhibits cell viability, cell cycle progression, and glutamine metabolism in TE14 cells. Mechanistic study indicates that NEDD4L regulates tumor progression through ubiquitination of c-Myc and modulation of glutamine metabolism. NEDD4L inhibits cell viability, cell cycle progression, and glutamine metabolism in ESCC by ubiquitination of c-Myc to decrease the expressions of GLS1 and SLC1A5. Our findings highlight the importance of NEDD4L/c-Myc signaling in ESCC.

## Introduction

Esophageal cancer is one of the leading causes of cancer-related death [1]. Esophageal squamous cell carcinoma (ESCC) is a common subtype of esophageal cancer [2]. ESCC incidence increases with aging. Alcohol abuse and tobacco usage are key risk factors [3]. Surgery remains the main strategy for treatment of ESCC at early stage. Concurrent pre-operative chemoradiotherapy is the only choice for advance ESCC [4]. However, the treatment outcome is unsatisfactory. Therefore, finding new therapeutics for ESCC is of great importance.

Deregulated glutamine metabolism has been proved to be a new hallmark of cancers [5]. Glutamine plays an important role in TCA cycle and redox homeostasis [6]. Enhanced glutamine uptake is mediated by several transporters, including solute-linked carrier family A1 member 5 (SLC1A5). Glutaminase (GLS), which catalyzes the conversion of glutamine to glutamate, is also involved in aberrant glutamine metabolism [7]. It has been shown that targeting GLS might be an alternative treatment for advanced breast cancer [8]. Data also support that targeting SLC1A5 can be used as a pre-operative therapy for esophageal cancer [9].

C-Myc deregulation is strongly associated with poor prognosis [10]. Activated c-Myc upregulates glutamine metabolism via up-regulation of GLS, making cancer cells dependent on high level of this amino acid to survive and proliferate [11]. c-Myc has been shown to act as a transcription factor which promotes ESCC cell proliferation [12]. Inhibition of c-Myc causes rapid tumor regression [13]. In most ESCC cases, c-Myc is positive in tumor tissues, and c-Myc overexpression is correlated with the degree of differentiation [14].

Protein ubiquitination is involved in various biological processes [15]. It is a enzymatic process that marks substrate proteins with ubiquitin, and the ubiquitinated proteins are subsequently degraded mainly via the ubiquitin-proteasome system (UPS) [16]. Dysregulation of ubiquitination and deubiquitination has been reported in different cancers [17]. For instance, increased expression of USP14 was observed in ESCC samples compared to that in their paired non-tumor tissues, and USP14 could be used for the prediction of unfavorable prognosis in ESCC [18]. Neural precursor cell expressed developmentally down-regulated 4-like (NEDD4L) is an ubiquitin protein ligase which binds and regulates various proteins [19]. Multiple proteins have been reported to be ubiquitinated by NEDD4L [19]. NEDD4L down-regulation is correlated with poor prognosis of patients in gastric cancer [20]. Although advances have been made in the study of NEDD4L, the role of NEDD4L in ESCC remains to be elucidated.

In the present study, we investigated the role of NEDD4L in ESCC, its association with the ubiquitination of c-Myc, and its relationship with the cell viability, cell cycle progression, and glutamine metabolism of ESCC cells. This study may provide a new therapeutic for the treatment of ESCC.

## Materials and Methods

### Bioinformatics

The RNA-Seq expression data for ESCC were extracted from the esophageal cancer dataset (162 esophageal cancer and 11 adjacent normal samples) in The Cancer Genome Atlas (TCGA, GDC V18.0, released on July 8, 2019), including 80 ESCC and 1 ESCC adjacent normal tissue. The data analysis was performed with R software using the DEGseq package. The threshold set for significant differences was log2|fold change|≥1 and *P*-value<0.05.

### Clinical samples

Twenty-five resected tumor and adjacent-normal samples were obtained from ESCC patients from our hospital between January 2018 and July 2020. These experiments were approved by the Ethics Committee of the Center Hospital of Karamay City and informed consents were received from all patients. ESCC tissue microarray analyses were performed by Outdo (Shanghai, China) and used for detecting NEDD4L and c-Myc expressions.

### Immunohistochemistry (IHC)

Samples were embedded, sectioned, and incubated with anti-NEDD4L antibody (ab46521; Abcam, Cambridge, UK) or anti-c-Myc antibody (ab32072; Abcam), followed by incubation with HRP-conjugated second antibody (Beyotime, Shanghai, China). After color development, the immunoreactivity was scored using the H-score system based on the percentage of positively stained cells (0,<5%; 1, 5%–25%; 2, 25%–50%; 3, 50%–75%; 4, >75%) and the staining intensity (0, negative; 1, weak; 2, moderate; 3, strong), which ranged from 0–12. ESCC patients were divided into low-expression group (H-score<4) and high-expression group (H-score ≥ 4).

### Cell culture

The human ESCC cell lines (TE1, TE11, TE14, and KYSE140) and normal esophageal epithelial cells HEEC were obtained from Beyotime (Shanghai, China) and cultured in RPMI-1640 (Sigma, St Louis, USA) supplemented with 10% FBS and 1% penicillin-streptomycin (Solarbio, Shanghai, China) at 95% humidity with 5% CO _2_ at 37°C.

### Gene overexpression and silencing

To overexpress c-Myc, the *c-Myc* gene was inserted into *Hin*dIII- and *Eco*RI-digested pcDNA3.1(+) vector (Addgene, Watertown, USA). Transfection was carried out for 6 h at 37°C using Lipofectamine 2000 (Invitrogen, Carlsbad, USA) according to the manufacturer’s protocol. To knockdown *NEDD4L*, 3 shRNAs (shNEDD4L#1, 5′-GAGCGACCCTATACATTTA-3′; shNEDD4L#2, 5′-GGGAAGTTGTTGACTCAAA-3′; and shNEDD4L#3, 5′-GCTCTTTGATTCAAAGAGA-3′) were annealed and cloned into *Age*I- and *Eco*RI-digested pLKO.1 lentiviral vector (Addgene). A non-targeting sequence (5′-GTAACGCGATATCTAGTCA-3′) was used as a shNC control. The cDNA encoding the full-length coding region of NEDD4L was subcloned into *Eco*RI- and *Bam*HI-digested pLVX-Puro lentiviral vector (Clontech, Palo Alto, USA) for constructing NEDD4L overexpression vector. Empty pLVX-Puro lentivirus plasmid was used as a vector control. To produce transducer plasmids, the recombinant lentivirus (1000 ng) were transfected along with the packaging plasmids psPAX2 (100 ng) and pMD2G (900 ng; both from Addgene) and amplified in 293T cells with Lipofectamine 2000 according to manufacturer’s protocol. Forty-eight hours after transfection, the recombinant lentivirus in the cell supernatant was collected by centrifugation at 5000 *g* for 5 min, and the purification and titration of recombinant lentivirus was performed as previously described [21]. ESCC cells were infected with the recombinant lentivirus-transducing units at an MOI of 20 in the presence of 8 μg/mL polybrene (Sigma) for 24 h at 37°C. Stable cells were selected using 3 μg/mL puromycin (Thermo Fisher Scientific, Waltham, USA) for four more days.

### Cell viability analysis

Cell viability was measured using Cell Counting Kit-8 (CCK-8) based on the manufacturer’s instructions. Briefly, cells were seeded in 96-well plates (3×10 ^3^ cells/ well) and incubated with CCK-8 (10 μL) for 1 h. Cell viability was determined by measuring the absorbance value (OD) at 450 nm with a microplate reader.

### Cell cycle analysis

After treatment, cells were collected by centrifugation at 1000 *g* for 5 min, and fixed with 700 μL of pre-cooled absolute ethanol. RNase A (1 mg/mL, 100 μL) was added to the fixed cells and incubated for 30 min in the dark. The resulting cells were further stained with 50 μg/mL of propidium iodide (PI; 400 μL) for 10 min, and then subject to analysis by flow cytometry on a FACScan flow cytometer (Becton Dickinson, Franklin Lakes, USA). Data analysis was then performed using the Cell Quest software (Becton Dickinson).

### Glutamine uptake

Glutamine uptake was measured using a Glutamine Assay kit (ab197011; Abcam) according to manufacturer’s protocol. According to the principle of glutamine conversion into glutamic acid and ammonia, the amount of glutamine was calculated by measuring the amount of ammonia. The relative glutamine uptake was normalized by the protein amount of each group.

### Quantitative RT-PCR

RNA was isolated using Trizol (Invitrogen) and reverse-transcribed into cDNA. Quantitative RT-PCR (RT-qPCR) was carried out using the SYBR Green kit (Qiagen, Hilden, Germany). Primers used are as follows: *NEDD4L*-F: 5′-CTCGGTGATGTGGATGTG-3′, *NEDD4L*-R: 5′-TTCGGCGTCCATGAGTAG-3′; *c-Myc*-F: 5′-TCCTGTCCGTCCAAGCAG-3′, *c-Myc*-R: 5′-ACGCACAAGAGTTCCGTAG-3′; and *β-actin*-F: 5′-TGGCATCCACGAAACTAC-3′, *β-actin*-R: 5′-CTTGATCTTCATGGTGCTG-3′. The relative mRNA expression was calculated by the 2 ^−ΔΔCT^ method. *β-Actin* was used as the internal control.

### Western blot analysis

RIPA buffer containing a protease inhibitor cocktail (Beyotime) was used to lyse cells. The cell was lysed for 30 min on ice. The extracted protein and Laemmli loading buffer were mixed at a 1:1 ratio before boiling for 5 min. Proteins were separated by 10% or 15% SDS-PAGE and transferred to PVDF membranes, The membranes were blocked with 5% skimmed milk for 1 h at room temperature, followed by incubation with primary antibodies against NEDD4L (ab46521; Abcam), c-Myc (ab32072; Abcam), GLS1 (ab156876; Abcam), SLC1A5 (ab237704; Abcam), or β-actin (#4970; CST, Beverly, USA) overnight at 4°C. Then corresponding HRP-conjugated secondary antibodies (Beyotime) were used for incubation at room temperature for 2 h. Membranes were then washed and visualized using an enhanced chemiluminescence kit (Millipore, Beverly, USA). Protein bands were analyzed using ImageJ software.

### Co-immunoprecipitation and ubiquitination assay

The cell lysates were incubated with anti-NEDD4L (ab240753; Abcam), anti-c-Myc (ab32072; Abcam), or control IgG (sc-2027; Santa Cruz, Santa Cruz, USA) for 1 h at 4°C. The mixture was then incubated with protein A/G-agarose (150 μg protein A; sc-2003; Santa Cruz) for 3 h at 4℃. After extensive wash, the immunocomplexes were eluted and subject to western blot analysis using anti-ubiquitin antibody (ab134953; Abcam) to detect the ubiquitination level.

### Protein stability assay

To evaluate protein stability, TE14 cells transduced with the indicated plasmids were treated with 0.1 mg/mL cycloheximide (CHX; Millipore) for indicated time intervals and harvested. The level of c-Myc protein was then determined by western blot analysis.

### Animal experiment

The 4–6-week-old male nude mice were purchased from the Shanghai Laboratory Animal Company (Shanghai, China). A tumor-bearing mice model was established by subcutaneously injected with 100 μL NEDD4L-overexpressing TE14 cells (5×10 ^6^ cells). Tumor growth was monitored twice a week. Finally, the mice were anesthetized by inhalation with 3% isoflurane and sacrificed by cervical dislocation. The mice were euthanized on day 33 and tumors were collected for immunofluorescence assay. All animal experiments were carried out in accordance with the Guidelines for the Care and Use of Laboratory Animals approved by the Ethics Committee of the Center Hospital of Karamay City.

### Immunofluorescence microscopy

Tissue sections were fixed, permeabilized, blocked and incubated with anti-Ki67 antibody (ab15580; Abcam) and Alexa Fluor 488-labeled Goat Anti-Mouse IgG (H+L) (A0423; Beyotime) antibodies. DAPI was applied to stain the cell nuclei. Tissue samples were finally examined under a confocal fluorescence microscope (Nikon Corp., Melville, USA).

### Statistical analysis

All experiments were conducted at least three times independently. Data were expressed as the mean±standard deviation (SD). GraphPad Prism 8.0.2 was used to analyze the data. A two tailed unpaired or paired Student’s *t* test was used to compare differences between two groups. A one way ANOVA followed by Tukey’s post-multiple test was used to compare differences among multiple groups. Kaplan-Meier method and Cox’s proportional hazards regression models were used to calculate overall survival and the differences were analyzed by a log-rank test. *P*<0.05 was defined as statistically significant.

## Results

### NEDD4L downregulation is correlated with poor prognosis in ESCC

**Figure 1:**
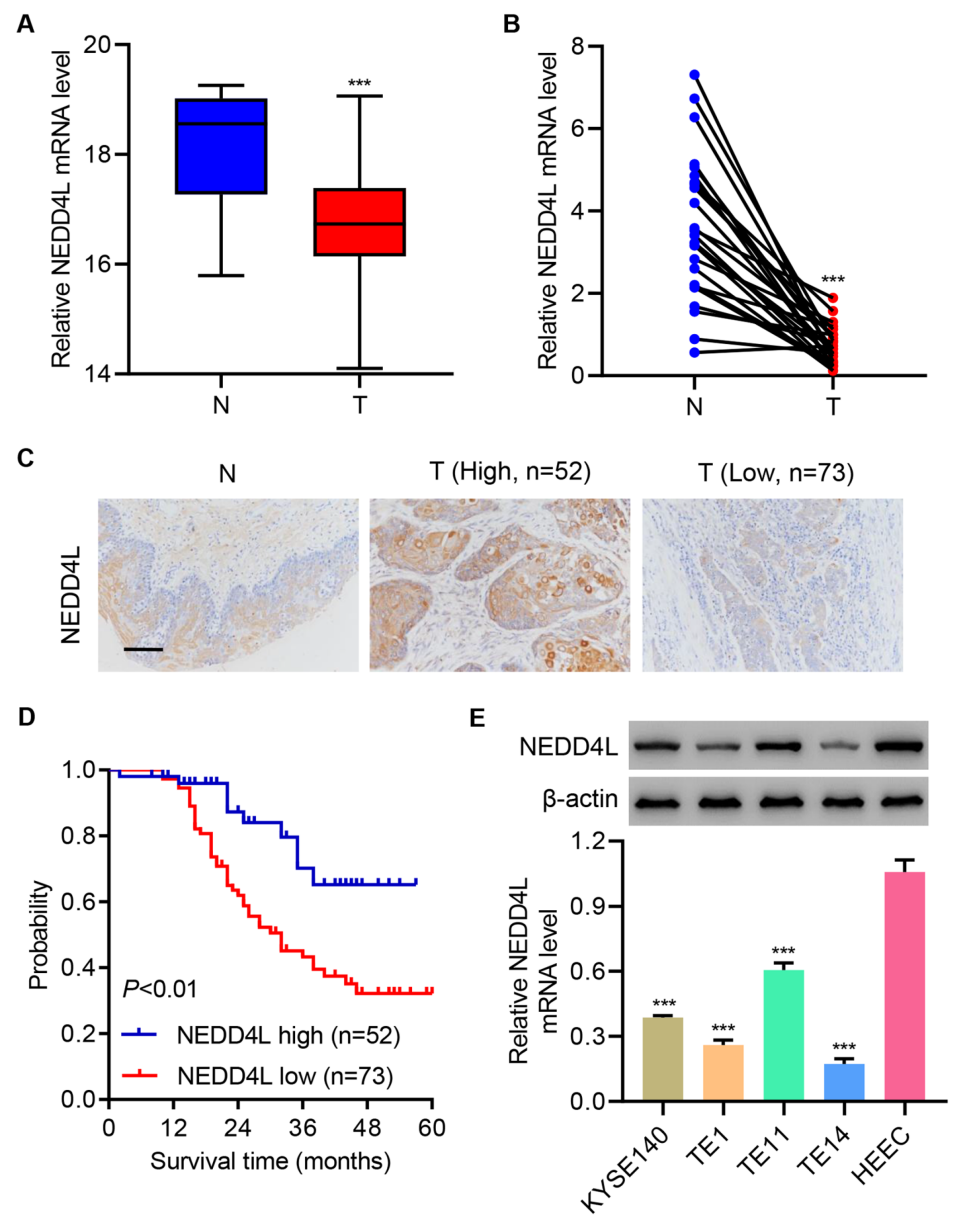

To study the role of NEDD4L in esophageal cancer, the level of NEDD4L in esophageal cancer samples was measured. The mRNA levels of NEDD4L in adjacent normal control (N) and esophageal cancer (T) tissues were measured. Results showed that NEDD4L was significantly down-regulated in esophageal cancer tissue compared with that in adjacent normal control tissue from both TCGA RNA-seq data ( Figure 1A) and clinical data ( Figure 1B). Based on immunohistochemistry (IHC) staining of NEDD4L, ESCC tissues were separated into NEDD4L-high expression group ( *n*=52) and NEDD4L-low expression group ( *n*=73) ( Figure 1C). Kaplan-Meier survival analysis showed that the overall survival rate was sharply decreased in NEDD4L-low expression group ( Figure 1D). The expressions of NEDD4L in ESCC cell lines (TE1, TE11, TE14, and KYSE140) were also significantly down-regulated compared with those in normal esophageal epithelial cells HEEC ( Figure 1E). Together, these data suggest that NEDD4L downregulation is correlated with poor clinical outcomes in ESCC.

### NEDD4L overexpression inhibits tumor growth *in vitro* and *in vivo*

**Figure 2:**
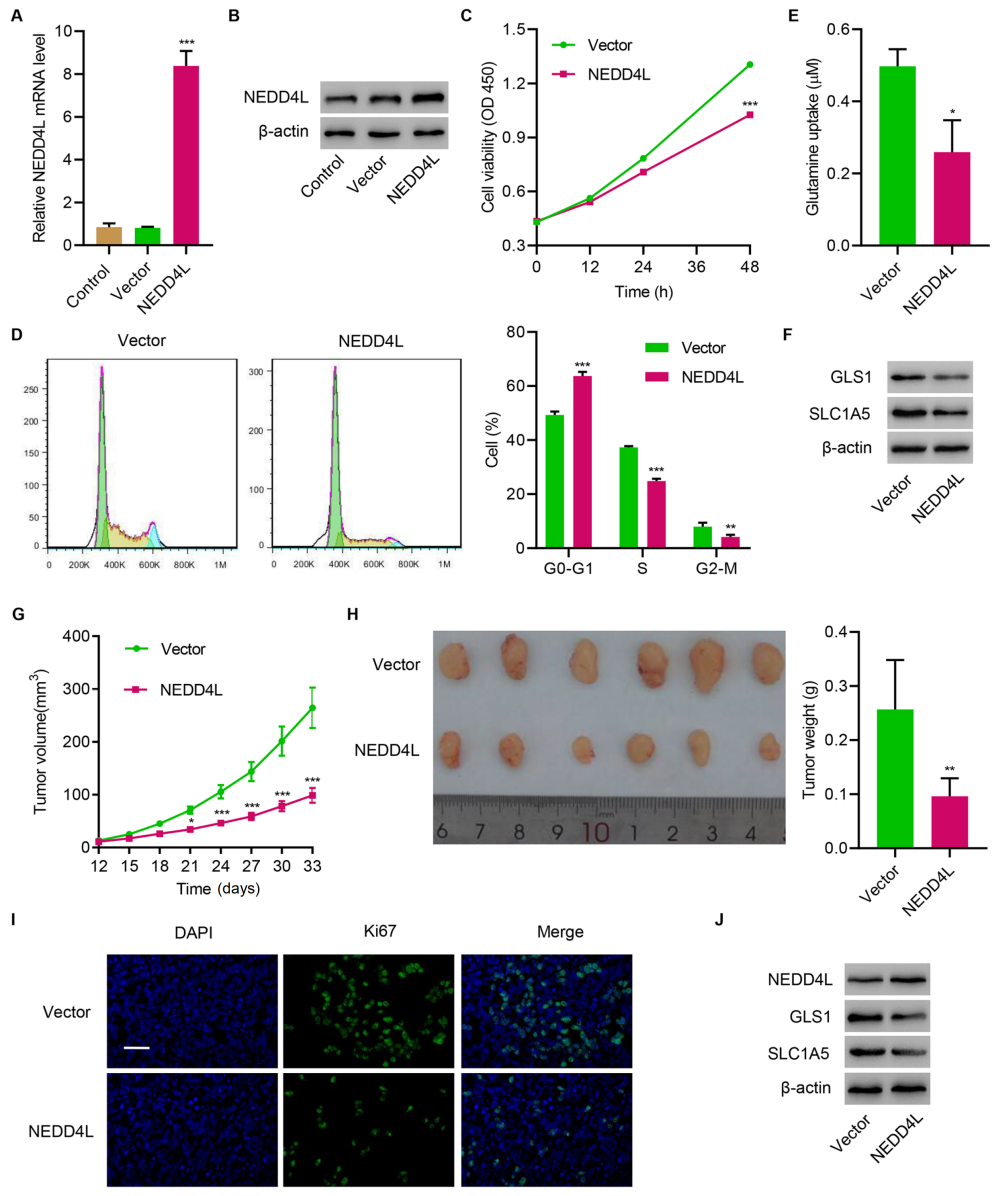

Next, NEDD4L was successfully overexpressed in TE14 cells ( Figure 2A,B). Overexpression of NEDD4L significantly decreased cell viability ( Figure 2C), caused significant cell cycle arrest ( Figure 2D), suppressed glutamine uptake ( Figure 2E), and inhibited the expressions of GLS1 and SLC1A5 ( Figure 2F). In vivo study showed that overexpressing NEDD4L also significantly inhibited tumor growth ( Figure 2G,H), suppressed tumor cell proliferation ( Figure 2I), and inhibited the expressions of GLS1 and SLC1A5 in tumor tissue ( Figure 2J). These results suggest that NEDD4L overexpression inhibits tumor growth *in vitro* and *in vivo*.

### NEDD4L interacts with and induces ubiquitination of c-Myc

**Figure 3:**
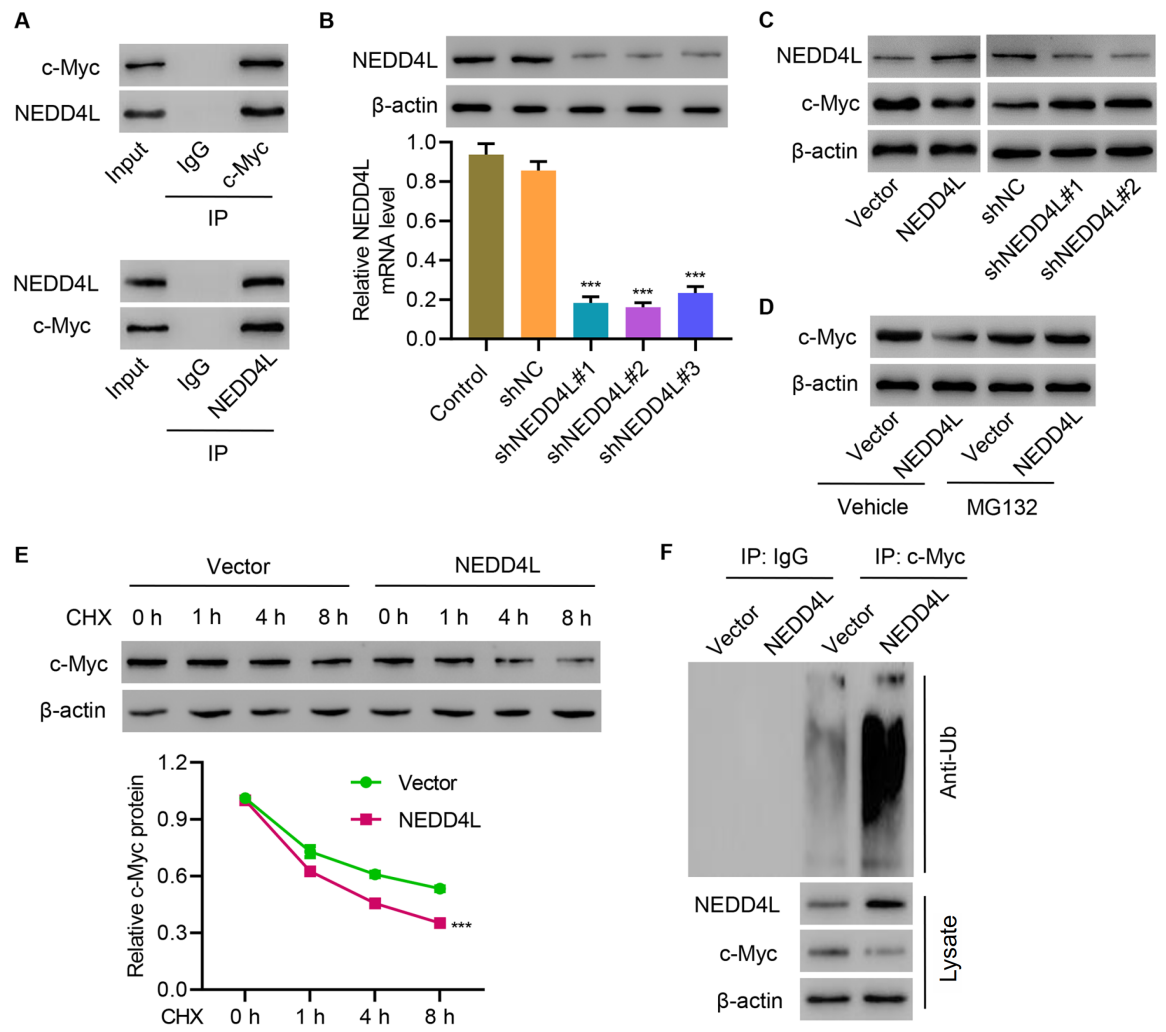

To investigate how MEDD4L is involved in regulating tumor growth, co-immunoprecipitation assay was performed. Results showed that NEDD4L interacted with c-Myc in TE14 cells ( Figure 3A). Then, NEDD4L was successfully silenced in TE11 cells ( Figure 3B). Silencing of NEDD4L significantly increased c-Myc expression in TE11 cells, while overexpression of NEDD4L significantly decreased c-Myc expression in TE14 cells ( Figure 3C). Administration of protease inhibitor MG132 reversed the inhibition of c-Myc expression in TE14 cells caused by NEDD4L overexpression ( Figure 3D). To further establish whether NEDD4L inhibits c-Myc stability, TE14 cells were treated with CHX and the half-life of c-Myc was determined. c-Myc stability was dramatically decreased in NEDD4L-overexpressing TE14 cells ( Figure 3E). These results demonstrate that NEDD4L destabilizes c-Myc. Mechanistic study showed that NEDD4L induced c-Myc ubiquitination to decrease its protein level in TE14 cells ( Figure 3F). These findings suggest that NEDD4L interacts with and induces ubiquitination of c-Myc.

### NEDD4L overexpression inhibits cell viability, cell cycle progression, and glutamine metabolism in TE14 cells through ubiquitination of c-Myc

**Figure 4:**
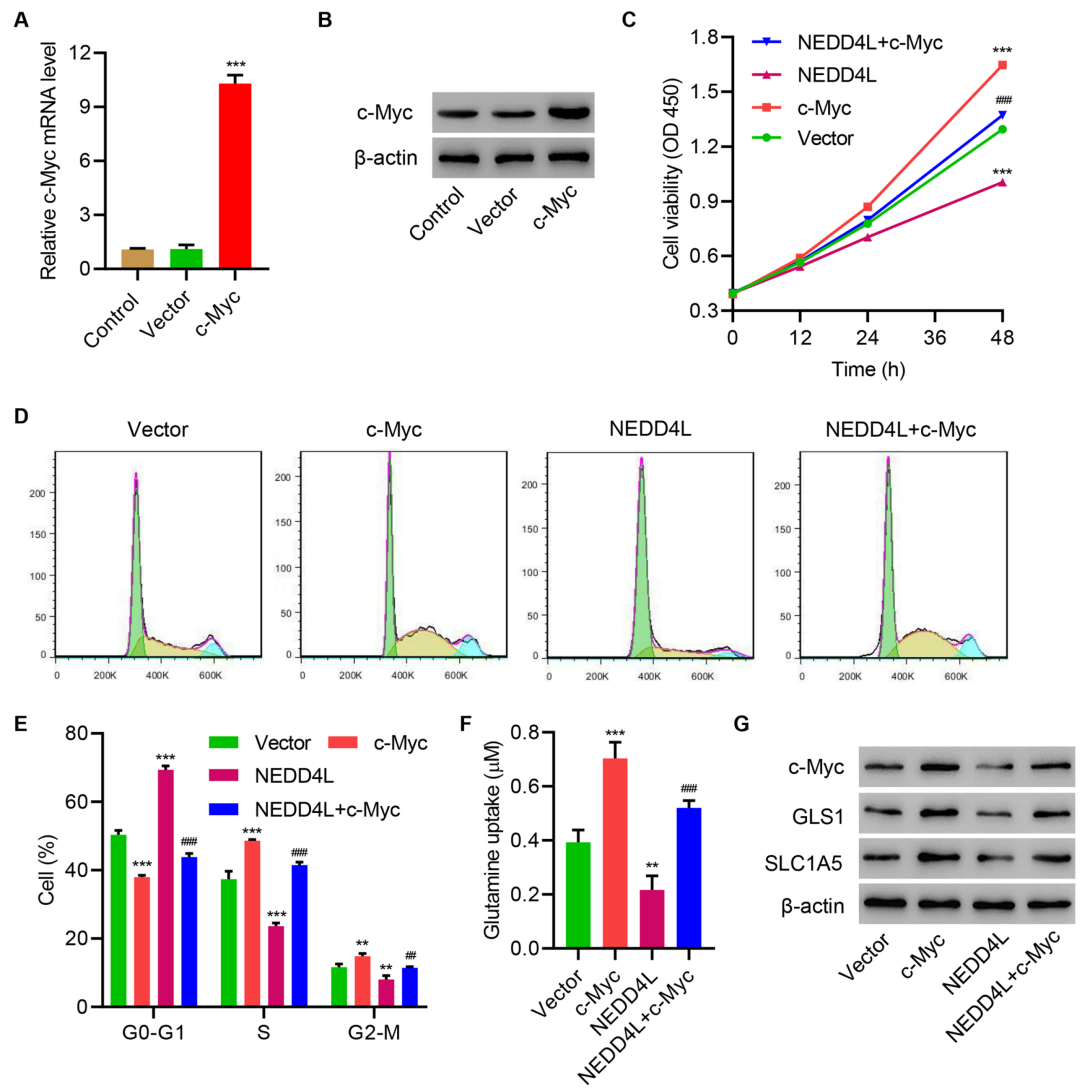

Next, c-Myc was successfully overexpressed in TE14 cells ( Figures 4A,B). Overexpressing c-Myc significantly increased cell viability ( Figure 4C), promoted cell cycle ( Figure 4D,E) and glutamine uptake ( Figure 4F), and increased the expressions of GLS1 and SLC1A5 of TE14 cells ( Figure 4G). More importantly, overexpressing c-Myc significantly ameliorated the effects induced by NEDD4L overexpression. These results demonstrate that NEDD4L overexpression inhibits cell viability, cell cycle progression, and glutamine metabolism in TE14 cells through ubiquitination of c-Myc.

### NEDD4L expression is correlated with c-Myc in patient samples

**Figure 5:**
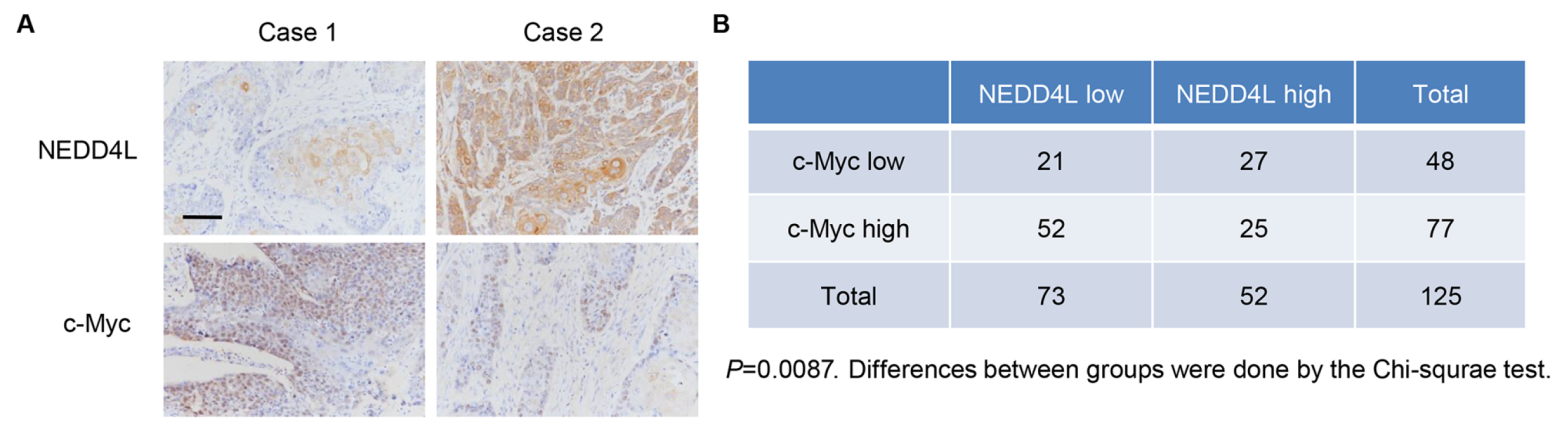

ESCC tissue microarrays were performed to measure the expressions of NEDD4L and c-Myc ( Figure 5A). Chi-square test indicated that NEDD4L was negatively correlated with c-Myc in ESCC samples ( Figure 5B).

## Discussion

In this study, we first analyzed NEDD4L expression in ESCC specimens and normal adjacent tissues, and found that NEDD4L was significantly decreased and its downregulation was significantly associated with poor prognosis. Further studies showed that overexpression of NEDD4L significantly decreased cell viability, arrested cell cycle, and inhibited the expressions of GLS1 and SLC1A5 to suppress glutamine uptake. Mechanistic study indicated that the effects of NEDD4L were mediated by ubiquitination of c-Myc. For the first time, our study indicated that NEDD4L inhibits cell viability, cell cycle progression, and glutamine metabolism in ESCC via ubiquitination of c-Myc.

The Myc family has 3 members, *i.e*., c-Myc, N-Myc, and L-Myc [22]. Expression of c-Myc protein is enhanced and deregulated in many human tumors. For instance, c-Myc is up-regulated in triple-negative breast cancer [23]. c-Myc down-regulation inhibits cell cycle and induces cell senescence of liver cancer cells [24]. It has also been shown that c-Myc promotes cell growth in ESCC [12]. In the present study, we showed that c-Myc overexpression promotes cell viability, cell cycle progression, and glutamine metabolism in ESCC. These findings highlight the importance of c-Myc in ESCC and may provide a potential therapeutic target for the treatment of ESCC.

The UPS is a mechanism for cells to get rid of proteins, which is catalyzed by E1 (ubiquitin activating), E2 (ubiquitin conjugating), and E3 (ubiquitin ligating) enzymes [ 25, 26]. As an ubiquitin protein ligase, NEDD4L regulates a number of membrane proteins via ubiquitination [19]. For example, Lee *et al*. [27] reported that NEDD4L inhibits autophagy via down-regulating ULK1 through ubiquitination. Tanksley *et al*. [28] demonstrated that NEDD4L suppresses colorectal cancer by ubiquitylating DVL2. NEDD4L also controls c-Myc stability in lung cancer cells [29]. In the present study, we proved that NEDD4L promoted c-Myc ubiquitination to decrease c-Myc protein level. These findings revealed a new role of NEDD4L/c-Myc ubiquitination in ESCC

Metabolic reprogramming is a hallmark of cancer cells [30]. But cancer cells can adapt to metabolic reprogramming [31]. Glutamine is involved in both biosynthesis and TCA cycle [ 32, 33]. GLS1, an enzyme which converts glutamine to glutamate, is involved in various cancers. GLS1 inhibition has been shown to inhibit tumor growth and metastatic progression [34]. Enhanced glutamine uptake is mediated by several transporters, including SLC1A5. Zhang *et al*. [35] reported that SLC1A5-dependent glutamine uptake is critical for the tumorigenesis of head and neck squamous cell carcinoma. Another study showed that SLC1A5 plays a key role in glutamine transport controlling the growth of lung cancer cells [36]. A recent study revealed that glutamine metabolism is upregulated in ESCC, which is indispensable for the development of tumors [37]. Oncogene c-Myc was reported to promote glutamine metabolism through directly binding to the promoter region of *SLC1A5* and *GLS1*, activate SLC1A5 and GLS1 by transcription, thereby increasing the expression levels of SLC1A5 and GLS1 [ 38, 39]. Our results indicated that overexpression of NEDD4L significantly inhibited the expressions of GLS1 and SLC1A5, leading to the suppression of glutamine metabolism, which may contribute to decreased cell viability and significant cell cycle arrest through ubiquitination of c-Myc. More importantly, suppression of glutamine metabolism caused by NEDD4L overexpression also inhibited tumor growth *in vivo* . These findings elucidate a novel role of NEDD4L/GLS1/SLC1A5 axis in glutamine metabolism, and broaden our understanding of NEDD4L/GLS1/SLC1A5 axis in the progression of ESCC, which may benefit the treatment of ESCC.

In should be noted that there are still some limitations in this study. For instance, only one cell line was used in this study, therefore future studies using more ESCC cell lines should be carried out to further confirm the results. A PDX mouse model is required to verify the functions of the NEDD4L/c-Myc/GLS1/SLC1A5 axis in ESCC progression. Nevertheless, our study demonstrates a novel role of the NEDD4L/c-Myc/GLS1/SLC1A5 axis in ESCC progression.
